# Nanofluidic Immobilization and Growth Detection of *Escherichia coli* in a Chip for Antibiotic Susceptibility Testing

**DOI:** 10.3390/bios10100135

**Published:** 2020-09-25

**Authors:** Jan F. Busche, Svenja Möller, Ann-Kathrin Klein, Matthias Stehr, Foelke Purr, Margherita Bassu, Thomas P. Burg, Andreas Dietzel

**Affiliations:** 1 Institute of Microtechnology, Technische Universität Braunschweig, 38124 Braunschweig, Germany; jan.busche@tu-bs.de (J.F.B.); svenja.moeller@tu-bs.de (S.M.); ann-kathrin.klein@tu-bs.de (A.-K.K.); f.purr@tu-bs.de (F.P.); 2 Lionex Diagnostics & Therapeutics GmbH, 38124 Braunschweig, Germany; mst@lionex.de; 3 Max Planck Institute for Biophysical Chemistry, 37077 Göttingen, Germany; margherita.bassu@gmail.com; 4 Department of Electrical Engineering and Information Technology, Technische Universität Darmstadt, 64283 Darmstadt, Germany

**Keywords:** optofluidic, nanofluidic, antibiotic resistance test, nano-grating, microfabrication

## Abstract

Infections with antimicrobial resistant bacteria are a rising threat for global healthcare as more and more antibiotics lose their effectiveness against bacterial pathogens. To guarantee the long-term effectiveness of broad-spectrum antibiotics, they may only be prescribed when inevitably required. In order to make a reliable assessment of which antibiotics are effective, rapid point-of-care tests are needed. This can be achieved with fast phenotypic microfluidic tests, which can cope with low bacterial concentrations and work label-free. Here, we present a novel optofluidic chip with a cross-flow immobilization principle using a regular array of nanogaps to concentrate bacteria and detect their growth label-free under the influence of antibiotics. The interferometric measuring principle enabled the detection of the growth of *Escherichia coli* in under 4 h with a sample volume of 187.2 µL and a doubling time of 79 min. In proof-of-concept experiments, we could show that the method can distinguish between bacterial growth and its inhibition by antibiotics. The results indicate that the nanofluidic chip approach provides a very promising concept for future rapid and label-free antimicrobial susceptibility tests.

## 1. Introduction

Antimicrobial-resistant (AMR) infections are one of the fastest growing threats for global healthcare. According to studies, about 700,000 people per year die around the world as a result of a multidrug resistant infection [1]. A cause for the development of multidrug-resistant bacteria strains is the prescription of antibiotics where they would not have been necessary. This happens when no test result is available in the short term. In this case, a broad-spectrum antibiotic must often be prescribed as a precaution [2,3,4]. This problem is aggravated when reserve antibiotics are used in therapy despite the fact that they would not have been required to help the patient. As a result, even those antibiotics can begin to lose their effectiveness to function reliably in future emergencies [5]. When antibiotics can no longer be used safely, a post-antibiotic era begins, in which all antibiotic-dependent therapies such as complex medical surgeries, chemotherapy and organ transplants can no longer be carried out [6].

In order to enable doctors to make decisions regarding the appropriate antibiotic treatment, quick antibiotic susceptibility tests (AST) are needed as point-of-care diagnostics. The available techniques for AST are divided into genetic and phenotypic methods, the latter of which study the behavior of the bacteria in the presence of various antibiotics [7]. While genetic methods provide results in around 90 min, they can only detect known genotypes and cannot detect unexplored resistance genes. This leads to the fact that existing resistances in infection-causing strains are not treated properly, which endangers the health of the patients [8]. Phenotypic methods, however, can investigate how the antibiotics directly affect the growth of the present bacteria. This information is most important for physicians and prevents false negative results with yet unknown resistance genes [9]. To date, these systems require a preculture (16–24 h) of the patient samples [10]. In the field of microfluidics, many approaches have recently been taken to improve the accuracy and measurement speed of phenotypic ASTs [11].

One limitation in the development of effective microfluidic ASTs is the cell loading procedure to accumulate bacterial cells in the systems. In one approach, 2.5 nL wells stacked with microbeads with a 5 µm diameter, acting as a filter, are used for pre-concentration of the bacterial sample. By measuring the redox potential, the response to antibiotics can be detected in 100 min [12]. Other systems rely on mixing liquid bacteria solution with agarose and subsequent cooling to capture the bacteria and measure their growth behavior with phase-contrast images in 1–4 h. However, these methods lack the ability to concentrate the bacterial sample within the chip [13,14,15]. In a multichannel system, channels with a cross section of 1 µm × 1 µm and a 300 nm gap in the end are used to capture the bacteria by filling up the channels from a feeding channel. The growth is detected individually for each channel, which requires the use of phase contrast microscopy and results in large data volumes that need to be analyzed [9,16]. A similar approach to capture bacteria in 2 µm × 2 µm channels with an 800 nm gap was used in a system where bacterial proliferation under the influence of antibiotics was measured by the electrical resistance in the channels [17]. These dead-end designs are prone to contaminants that can obstruct the flow in the channels, preventing the transport of nutrients and antibiotics to the bacteria [18]. Other methods use optical trapping [19], droplet microfluidics [20,21,22], dielectrophoresis [23] or antibodies bound to the sensor surface [24] for immobilization. All these methods have in common that the effort to immobilize bacteria is high, because either an additional laser, elements for droplet generation and droplet readout, or molecular recognition elements like antibodies, which are not available for all bacteria, are required.

To avoid complex immobilization procedures, the steps of trapping and susceptibility testing of bacteria need to be integrated in a single device. Therefore, a hydrodynamic bacterial cell-trapping principle which has been recently been reported [24] is now implemented in the form of a regular nanogap array to allow readout with an interferometric method. Here, we present an optofluidic chip which can immobilize bacteria in an optical asymmetric grating to measure the proliferation of bacteria with high sensitivity by interferometry based on optofluidic concepts that, in the past, had been developed to measure surface bound molecules [25,26]. This study describes the fabrication and characterization of the optofluidic nanosystem and shows the applicability of this approach in future ASTs.

## 2. Materials and Methods

### 2.1. Nano-Grating Chip Concept

**Figure 1:**
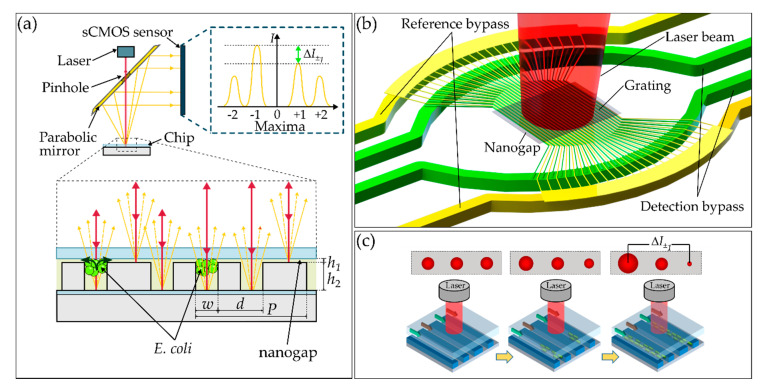
(**a**) Schematic drawing of the measuring principle with a cross-section of the chip and captured bacteria in the detection channels with a height of *h*_2_ = 4 µm, a width of *w* = 3 µm, a displacement of *d* = 6 µm and a period of *P* = 15 µm. Bacteria are captured and cultivated at the 590 nm (*h*_1_) gap. Green arrows in the cross-sectional view indicate the fluid flow. (**b**) Schematic drawing of the microfluidic channels on the chip (yellow and green) and the collimated laser beam (red) directed perpendicular onto the grating. (**c**) Schematic illustration of the change in the difference of the intensities of the maxima (Δ*I_±_*_1_) over time as growing bacteria are captured in the detection channels by a pressure loss over the nanogap.

A schematic of the nanofluidic chip with the optofluidic diffraction grating is shown in Figure 1a. The fluidic grating consists of two arrays of microchannels which are placed in interdigitating fashion to build up an asymmetric grating of measurement and reference channels with a channel width of *w* = 3 µm, a displacement of *d* = 6 µm and a period of *P* = 15 µm. Bacteria are captured and cultivated at the 590 nm (*h*_1_) wide cross flow filtration gaps in the middle of the grating in the 4 µm (*h*_2_) deep detection channels, which are shown in green in Figure 1b. The hydrodynamic capturing is realized by a pressure difference between adjacent channels. The reference channels allow a comparison with optical properties of the bacteria-free medium. The asymmetric grating results in a diffraction pattern when irradiated with a collimated laser beam (red). Only when bacteria are located in the detection channels, and, therefore, the refractive index in these channels is different from the reference channels, is a signal change detected on an sCMOS (scientific complementary metal-oxide-semiconductor) sensor, resulting in a difference in the intensities (Δ*I*_±1_). The derivation and calculation of the signal have been described previously and can be summarized in Equation (1) [27]. The common mode rejection of this measurement principle was shown before [26].

(1) $$S_{1}=\frac{I_{1}-I_{-1}}{I_{1}+I_{-1}}$$

### 2.2. Optofluidic Measurement Setup

**Figure 2:**
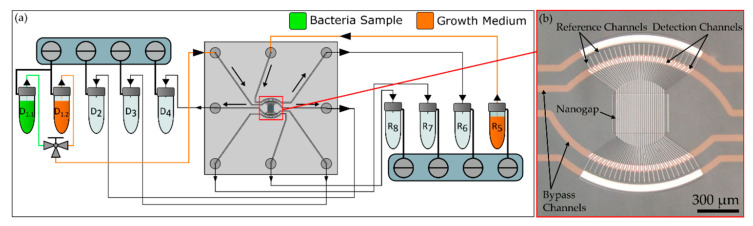
(**a**) Schematic of the fluidic setup with eight pressure controllers and sample reservoirs for the detection channels (*D*_1.1_ to *D*_4_) and reference channels (R_5_ to R_8_) connected to the nanograting chip and a three way valve to switch between capture and growth mode. (**b**) Microscopic top view image of the nanofluidic chip with the grating in the center surrounded by the bypass channels.

As illustrated in Figure 1a,b a values, the signal intensities of the maxima (*I_±m_*) are obtained. A pinhole (diameter = 3 mm) in the collimated single mode laser beam (Thorlabs LPS-635-FC with collimator CFC2-A, *λ* = 635 nm, waist diameter = 360 µm (1/e^2^ width)) is directed onto the grating by a parabolic mirror, which leads to the generation of a diffraction pattern on a sCMOS sensor (panda, PCO AG, Kelheim, Germany). By local integration of the gray mirror that directs the diffracted light to the camera suppresses the intense central maximum. The fluidic flow and the bacterial loading of the chip were realized by eight pneumatic controllers (MFCS-EZ, Fluigent, Le Kremlin-Bicêtre, France), which were connected by polytetrafluoroethylene (PTFE) tubes (ID = Ø 180 µm, Techlab, Germany) to eight sample vials (*D*_1.1_ to *R*_8_) (Biozym Scientific GmbH, Hessisch Oldendorf, Germany). A 5 µm PTFE membrane (Whatman, Maidstone, UK) between the tube outlets and the chip inlets acted as a filter to prevent clogging. To switch between bacterial capturing and growth mode, a three-way valve (2-Switch, Fluigent, Le Kremlin-Bicêtre, France) was used, as shown schematically in Figure 2a. The fluidic setup is integrated into the optical setup and is surrounded by an opaque housing, which was heated to 37 °C during the experiments by an incubator (ES-20, Grant Instruments Ltd., Cambridge, UK).

### 2.3. Chip Fabrication

**Figure 3:**
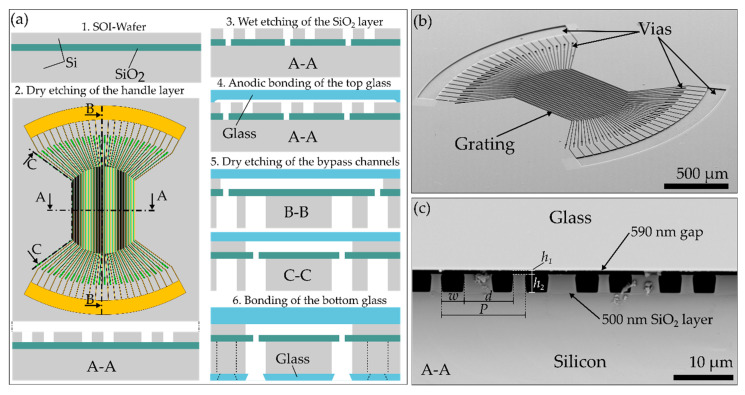
(**a**) Schematic of the fabrication steps for the nanofluidic grating chip with a top view for the first step and cross-sectional views for the following steps. (**b**) Tilted SEM top view of the optical grating after the first 4 µm deep etching into the silicon device layer prior to bonding. (**c**) SEM cross-sectional view of the nanogap area after anodic bonding of the top glass with gap width *h*_1_, channel height *h*_2_, channel width *w*, displacement *d* and period *P*.

The nanograting chip used in this study was fabricated with microfabrication techniques, as shown in Figure 3a. First, the optical grating was dry-etched 4 µm deep into the device layer of a 4-inch silicon-on-insulator (SOI) wafer (DISCO HI-TECH GmbH, Kirchheim, Germany) using photolithography and an adapted mixed gas dry etching process (STS Multiplex, STS, Newport, UK) [28,29]. This etch process leads to smoother sidewalls compared to sidewalls obtained by fast etching with gas-switching processes. The fluidic channels forming the optical grating have a length between 910 and 1016 µm depending on their position in the grating. The grating consisted of 24 detection and 24 reference channels. The 500 nm thick buried oxide layer beneath the device layer acted as a planar etch stop. A dip in buffered hydrofluoric acid (BHF, 7%) at room temperature for 15 min opened up the buried oxide layer at every end of the microfluidic channels in the device layer. This led to the opening of vias with a width of 8 µm at the end of the detection channels. The ends of the reference channels were connected by only one via per end. Thereafter, a 200 µm thick Borofloat 33 wafer (Plan Optik AG, Elsoff, Germany) was etched 590 nm deep with 7% BHF for 7 min to generate the nanogap area. After a cleaning step in piranha solution (1:3 H_2_O_2_:H_2_SO_4_), the Borofloat 33 and the SOI wafers where anodically bonded (450 °C, 700 V, 750 mbar, 1 h) to seal the structured top sides. To minimize the fluidic volume needed for an assay and reduce the risk of footing in the next dry-etching step, the handle layer of the SOI wafer was thinned down to a thickness of 50 µm. Subsequently, 50 × 50 µm deep and wide microfluidic bypass channels were dry-etched with a gas switching process from the backside into the handle layer until the buried oxide layer was reached. This step established a fluidic connection to the grating in the device layer. Figure 3a,b show a SEM image of the top of the optical grating and a cross-section of the nanogap area. Due to the reduced thickness of the handle layer, no footing could be detected at the buried oxide layer, which acted as a planar etch stop. To seal the backside of the system, a 500 µm thick Borofloat 33 wafer was bonded to the bottom of the wafer (450 °C, 900 V, 750 mbar, 1 h). Prior to the anodic bonding, eight trough-holes (700 µm in diameter), acting as fluid in- and out-let ports, were drilled into the Borofloat 33 wafer by femtosecond laser ablation (Pharos, Light Conversion, Vilnius, Lithuania). After bonding, the wafers were diced into 10 × 10 mm chips.

### 2.4. Bacterial Strain and Growth Medium

For all experiments, an *Escherichia coli* DH5-α strain was used. To maintain sterility, the plasmid pPS858 encoding the genes *bla* (beta-lactam-resistance) and *aacC1* (gentamicin-resistance) was introduced to the cells, and 100 µg/mL carbenicillin and 10 µg/mL gentamicin-sulfate were added to TB (Terrific Broth) growth medium. Bacterial suspension containing 10^6^ *E. coli* cells/mL was used as loading sample. To test growth inhibition, 200 µg/mL kanamycin was added to the TB growth medium. To avoid clogging of the microchannels, media and bacterial suspensions were filtered with 0.2 µm and 5 µm syringe filters (Whatman, Maidstone, UK) prior to experiments. All growth experiments in this study were carried out at 37 °C.

### 2.5. Determination of Growth Rates

Collected data were analyzed and illustrated using Origin 2018 software (OriginLab Corporation, Northampton, MA, USA). For easier comparison, the signals acquired from optofluidic growth experiments were normalized with their associated baseline. In case of strong signal spikes, due to air bubbles or similar interruptions, corresponding data were excluded from further calculations. To calculate growth rates, the logarithm of the signal S_1_ was plotted over time. Linear regression was applied to the curve 30 min after beginning for 2.5 h. The slope corresponds to the refractive index change and equals the bacterial growth rate in min^−1^.

## 3. Results and Discussion

### 3.1. Device Characterization

**Figure 4:**
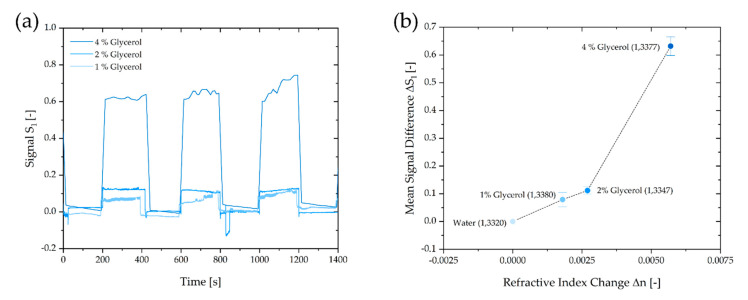
(**a**) Plot of the signal S_1_ as a function of the measuring time. With the introduction of glycerol solution, a sudden change in S_1_ is observed. As soon as distilled water enters the grating, the signal drops back to the initial value. (**b**) Plot of the mean signal difference ΔS_1_ over the refractive index change Δ*n*.

To characterize the fluidic chip, we conducted calibration measurements with different glycerol dilutions in deionized DI water with concentrations of 0 %, 2% and 4% (*w*/*w*), which represents a refractive index range of *n* = 1.3300–1.3377. The refractive index depends on the concertation of the solution [26]. Prior to the measurement, the detection and the reference channels were filled with DI water. After 5 min, the glycerol dilution was filled into the detection channels, and the signal S_1_ showed the change in the refractive index. This procedure was performed three times with each concentration to calculate a mean signal difference ΔS_1_ by averaging the three values for each concentration. After the measurements with glycerol were completed, the baseline could be reached again by rinsing with DI water in each experiment. The results are shown in Figure 4a,b.

### 3.2. Bacterial Capture Experiments

**Figure 5:**
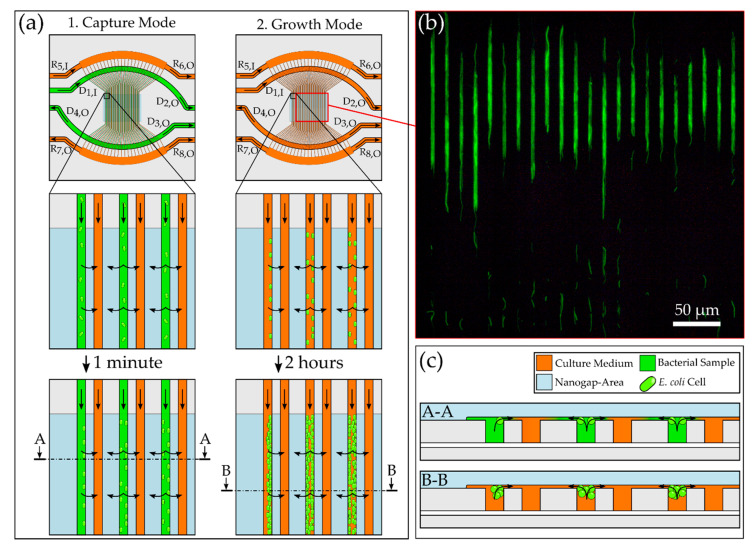
(**a**) Schematic illustrating the flows from the vials *D*_1–4_ and *R*_5–8_ in capture mode to load bacteria into the grating and the growth mode in order to observe the growth behavior of immobilized *Escherichia coli*. (**b**) Fluorescent image of bacteria immobilized at the nanogap area and incubated for 240 min. (**c**) Schematic cross-sectional view of the nanogap area.

A bacterial dilution of 10^6^ *E. coli* per mL in TB medium was used to validate whether the nanofluidic chip can be used to detect changes in refractive index due to bacterial capture and growth. To obtain useful pressure settings at the in- and out-lets, experiments with fluorescent *E. coli* were conducted using real-time fluorescent microscopy (Axiovert, Zeiss, Germany). The microscopic setup for these experiments has been described in our previous work [29]. The goal was to find conditions which result in captured bacteria at the nanogap area without damaging them or hindering their growth. The finally selected pressures used in this study are summarized in Table 1. With these pressure settings, a flow velocity of 7.2 µL from the sample reservoir inlet *D*_1,I_ to the outlet *D*_1,O_ could be realized. The pressure differences were chosen to be below the pressures at which shear stresses occur that affect the viability of the bacteria [29,30]. Figure 5a shows the procedure of loading the chip with a bacterial sample in capture mode, where the chip is filled with bacteria for later growth detection (see Supplementary Materials Video S1). A pressure difference between the detection and the reference channels led to immobilization of bacteria at the nanogap. The loading time was 26 min. Figure 5b shows a fluorescent image of the grating after successful loading and 3 h of growth of *E. coli*. The pressures at the in- and outlets led to a loading of bacteria beginning in the front area of the grating because pressure drops across the nanogap are highest in this area.

### 3.3. Growth Experiments with Fluorescence

**Figure 6:**
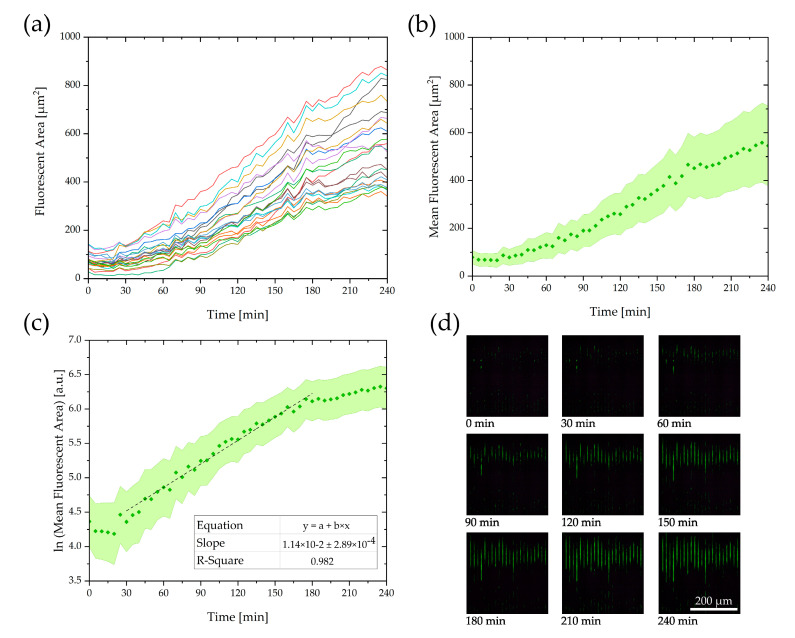
(**a**) Plot of the fluorescent area F of the individual channels over time. (**b**) Mean value M_F_ of the fluorescent area over time. (**c**) Plot of the logarithm of the averaged area M_F_ over time with the linear fit to determine the bacterial growth rate. (**d**) Fluorescence images taken during the growth experiments in intervals of 30 min for 4 h.

To determine if the bacteria are immobilized and grow in the correct position in the nanograting, growth experiments were performed with a fluorescence microscope and time lapse imaging as described in our previous work [29]. Graphical measurement of the fluorescent area in the growth channels resulted in fluorescent area-over-time plots per channel. Averaging over all channels allowed calculation of a bacterial growth rate for the whole grating. Figure 6d shows fluorescent images with time intervals of 30 min after the loading of the chip was completed. The images show that the bacteria started to grow from the beginning of the nanogap area to the middle of the grating and were successfully held back by the gaps for 4 h. In Figure 6a,b, the averaged fluorescent area is plotted over time. Figure 6c shows a plot of the logarithm of the averaged area with a linear regression. The slope corresponds to a bacterial growth rate of 0.0114 min^−1^, resulting in a doubling time of about 61 min. The experiments with green fluorescent protein GFP-labeled bacteria could show that for the complete duration of the experiments, no *E. coli* enter the gaps or reference channels. In contrast to previous studies, our system does not have a chemotaxis-inducing gradient [31] or a high-pressure difference [32] that forces the bacteria into the gaps.

### 3.4. Growth Detection with Diffraction Signal Measurement

**Figure 7:**
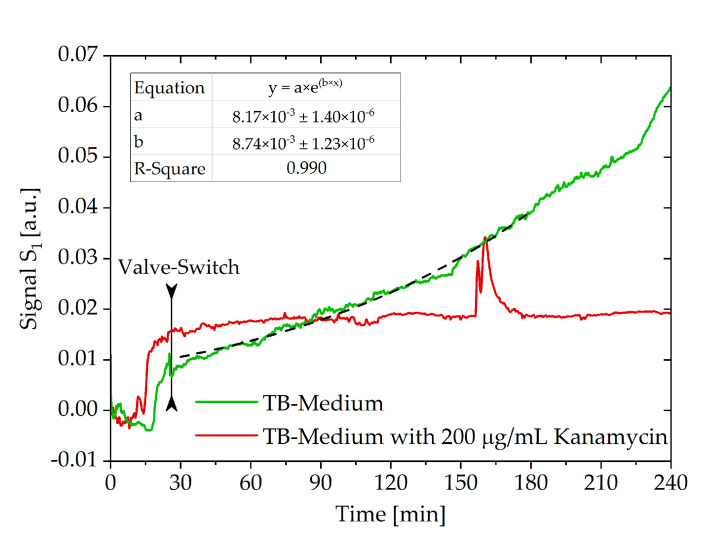
Time course of the diffraction signal of growth experiments with and without the addition of 200 µg/mL kanamycin with an exponential fit to determine the bacterial growth rate in the experiment without kanamycin.

To test if the optofluidic nanograting chip can detect the influence of antibiotics on the growth of immobilized bacteria, growth experiments with evaluation of the diffraction signal were carried out. Culture medium and bacterial dilution containing 10^6^ cells/mL were connected to the same chip inlet trough a three-way-valve. Pure culture medium was connected to the reference inlet. After filling the connecting tubes completely with medium by using 300 mbar of pressure for 1 min, pressures according to Table 1 were applied. For 10 min, the signal was recorded without loading of bacteria to obtain a stable signal and a basis for signal normalization. To load the chip with *E. coli* and start the experiment, the three-way valve was switched to the bacteria reservoir *D*_1.1_ and switched back to growth medium after 26 min, resulting in a sample volume of 187.2 µL. This resulted in a theoretical cell count of 187.2 × 10^3^ *E. coli* used for the experiment. The pressures at the pressure controllers were not changed during this time. This allowed for a bolus of bacterial sample to enter the chip with a short delay of 1 min without overloading the nanograting with bacterial cells. After switching the valve back to the pure media reservoir *D*_1.2_ for growth mode, the diffraction signal was observed continuously for 4 h. The growth experiments were conducted without and with effective antibiotics, for which 200 µg/mL kanamycin was added to the growth medium. Figure 7 shows the obtained signal courses over time, which are generated by laser diffraction at the nanograting consisting of detection and reference channels. Thus, the signal averages the refractive index change over all microchannels in the grating. In the beginning, the rise of the signal is caused by the loading of the chip with bacteria. After switching the valve to growth mode, a subsequent exponential signal increase is observed, indicating bacterial growth in the experiment without kanamycin. Plotting the logarithm of the signal over time resulted in a bacterial growth rate of 0.0090 min^−1^, which corresponds to a doubling time of about 79 min. In contrast, in the experiment with added kanamycin, the signal remained at a constant low level over 4 h. Due to the kanamycin sensitivity of the bacterial strain used here, this can be correlated with no bacterial growth and, therefore, no refractive index change inside the optical grating. If the standard deviation in the measurement curve is below three times the standard deviation of TB medium with 200 µg/mL kanamycin (60–90 min, SD = 3.35 × 10^−4^), this is considered as no growth. Errors or sudden peaks of the signal courses presumably correspond to air bubbles or other major movements inside the nanograting, but as shown in Figure 7, they do not affect the growth measurement permanently.

## 4. Conclusions

In this study, we demonstrated nanofluidic bacteria capturing and interferometric measurement of cell proliferation in a novel fluidic chip with nanofluidic cell trap arrays made from SOI wafers and femtosecond laser-structured glass closures. The adapted dry-etching process proved to be suitable for the production of the optical grating. By using a SOI wafer, two interdigitated gratings that were connected by horizontal nanofluidic gaps could be manufactured. Due to the use of glass and silicon, the chips can be completely cleaned and reused after experiments. Fluorescence experiments showed that the capturing of bacteria inside the nanograting takes place in the correct location and demonstrated bacteria grow with a doubling time of 61 min. Using our new interferometric method, we were able to determine cell growth in the nanograting with a doubling time of 79 min within a separate experiment. In agreement with other studies in comparable silicon-based microchips [31], the measured doubling times exceed the value of about 20 min known in common laboratory cultivation of *E. coli* [33]. This may be explained by limited nutrient or oxygen supply in restricted spatial dimensions. Nevertheless, the sensitivity of the measurement is sufficient to reliably detect cell proliferation in less than 4 h. The obtained signal revealed a comparable growth rate and doubling time as in the fluorescent experiments. This suggests a linear relationship between the interferometric signal and the biomass contained in the nanograting. The optofluidic setup including the three-way valve enables convenient switching between capture and growth modes, which ensures the detection of growth without further loading during the subsequent measurement. The needed sample volume for conducting the diffraction measurements was 187.2 µL. Thus, this design enables the capture of *E. coli* in combination with a label-free phenotypic growth detection. With an improved measurement setup, the presented detection principle could prove useful in future point-of-care devices for antimicrobial susceptibility testing (AST) where no overnight culture and labeling of the bacteria is possible. It has been shown that a similar optical setup can be realized for protein detection based on a smartphone [25]. In future studies, we plan to investigate if a dependence of the antibiotic concentration can be correlated with the measuring signal of the setup. To perform multiplexed measurements with different antibiotics and different antibiotic concentrations, several of these chips will have to be arranged in an automated test cartridge for alternating laser detection to perform time lapse measurements. Further experiments will also be conducted to investigate if this detection principle is suitable for high-throughput measurements of the minimal inhibitory concentrations of antibiotics.

## Figures and Tables

**Table 1:** Pressure settings at inlets and outlets of the microchip for growth experiments in capture and growth mode.

Pressure Applied (mbar) at the Inlets and Outlets							
*D*_1,I_	*D*_2,O_	*D*_3,O_	*D*_4,O_	*R*_5,I_	*R*_6,O_	*R*_7,O_	*R*_8,O_
380	350	350	350	200	180	180	180

## Supplementary Materials

The following are available online at https://www.mdpi.com/2079-6374/10/10/135/s1, Video S1: Inside view of the optofluidic microchip with bacterial loading and capturing.
